# Intraoral presentation of multiple malignant peripheral nerve sheath tumors associated with neurofibromatosis-1

**DOI:** 10.4103/0973-029X.80025

**Published:** 2011

**Authors:** Mahija Janardhanan, S Rakesh, RB Vinod Kumar

**Affiliations:** *Department of Oral Pathology and Microbiology, Amrita School of Dentistry, Kochi, Kerala, India*

**Keywords:** Malignant peripheral nerve sheath tumor, neurofibramotosis-1, oral

## Abstract

Neurofibromatosis-1 (NF-1) is a relatively common autosomal dominant disease characterized by multiple cutaneous fibromatoses and café au lait spots. It is associated with the mutation of *NF-1* gene, a tumor suppressor gene located on chromosome 17q11.2. Hence, it can be considered as a familial cancer predisposition syndrome in which the affected individuals are at increased risk of developing malignancies. Intraoral neurofibromas associated with NF-1 are quite common, but the occurrence of malignant peripheral nerve sheath tumor (MPNST) in the oral cavity is very rare. Oral MPNST can occur either *de novo* or by malignant transformation of neurofibromas or very rarely can represent a metastatic lesion. Here, we present a case of MPNST involving the maxillary region, in a patient with NF-1. Since MPNST often creates a diagnostic dilemma, histopathologic criteria for the diagnosis of MPNST are also discussed.

## INTRODUCTION

The term “neurofibromatosis” refers to a group of genetic disorders that primarily affect the cell growth of neural tissues. Neurofibromatosis-1 (NF-1) is the most common type and accounts for about 90% of all cases. It is one of the frequent genetic diseases with a prevalence of 1 case in 4000 births.[1] The expressivity of NF-1 is extremely variable with manifestations ranging from mild lesions to several complications and functional impairment. The syndrome is characterized by the presence of *café au lait* pigmentation on the skin, cutaneous neurofibromas, central nervous system tumors, pigmented hamartomas of the iris and skeletal abnormalities. Oral neurofibromas are seen in 72% of the NF-1 patients.[2] One of the most feared complications of NF-1 is the malignant transformation of benign tumors. Malignant peripheral nerve sheath tumor (MPNST), the principal malignancy of peripheral nerve origin, though rare in general population, occurs with excessive frequency among patients with neurofibromatosis. It represents 10% of all soft tissue tumors, with about half of such cases occurring in patients with neurofibromatosis.[3] MPNSTs are usually seen in the extremities and trunk and their occurrence in head and neck region is very rare.

We report a case of multiple MPNST in a patient with NF-1, who presented with a swelling on the right maxillary region. Other sites involved include mediastinum, scalp and upper back region and all the lesions developed in a short span of 6 months.

## CASE REPORT

**Figure 1:**
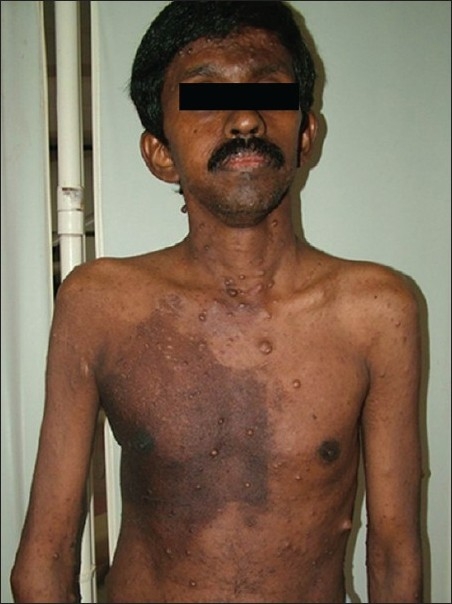
Cutaneous nodules distributed over the entire body. Note the large pigmented macule in the right chest

**Figure 2:**
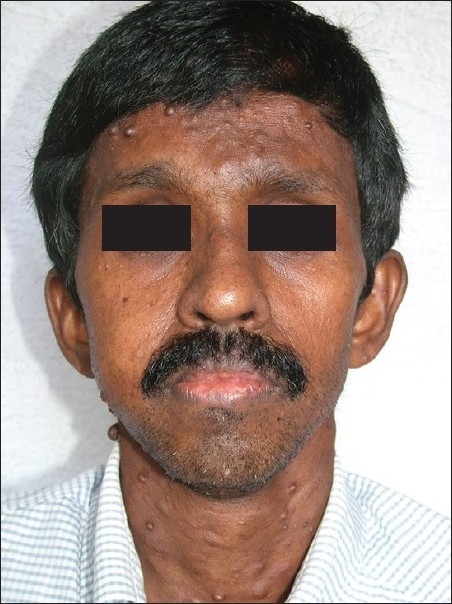
Cutaneous nodules on the face. Facial asymmetry due to the intraoral swelling can also be appreciated

**Figure 3:**
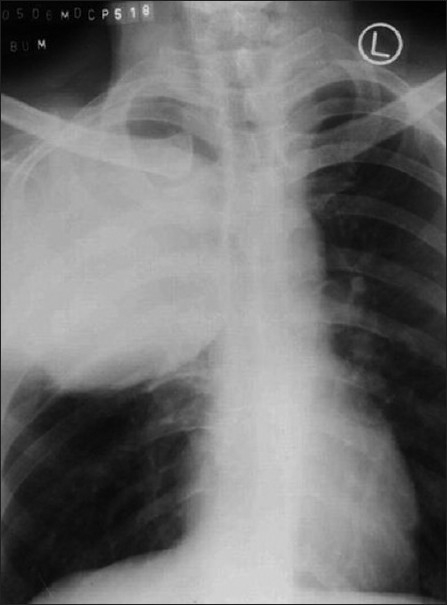
Chest radiograph showing the right mediastinal tumor

**Figure 4:**
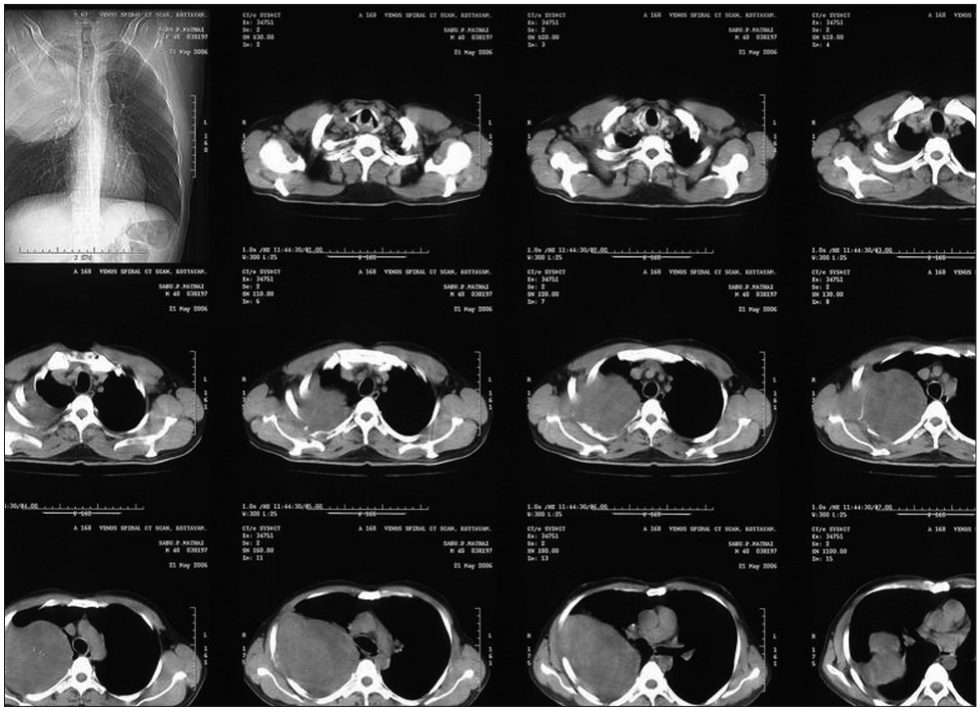
Spiral CT chest showing a large, well-defined soft tissue density mass lesion in the posterior aspect of the right upper hemithorax. The lesion is found to be extending to the chest wall with erosion of right upper ribs

**Figure 5:**
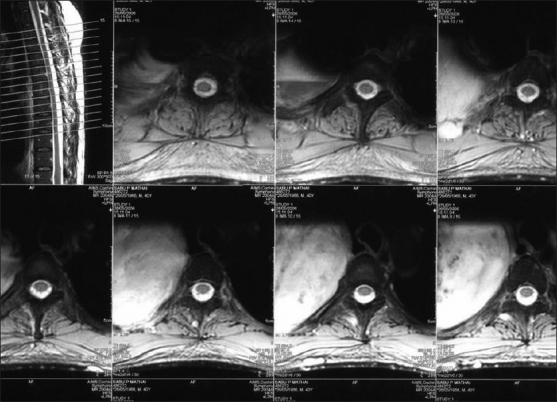
MRI of the thoracic spine sagittal T1 T2 level showing a large mass in the right thorax of which the medial border is adjacent to the thoracic vertebra. No evidence of vertebral encasement or intraspinal extension seen

A 40-year-old male patient, a known case of NF-1, reported to our hospital with the complaint of a swelling on the right maxillary region in relation to upper back tooth. The swelling, initially noticed 3 weeks back, was small in size, but grew rapidly to reach the present size. He also gave the history of dull aching pain associated with the swelling. His past medical history revealed that multiple cutaneous nodules seen on the entire body [Figures 1 and 2] were present since he was 13 years old. He was evaluated for the complaint of pain in the right chest 4 months back, following which chest X-ray, computed tomography (CT) and magnetic resonance imaging (MRI) scan were taken. The imaging studies revealed the presence of a mediastinal tumor which was later diagnosed as MPNST [Figures 3–5]. Since the lesion was inoperable, he was subjected to radiotherapy. His family history was noncontributory.

On general examination, the patient was poorly built and nourished. No pallor, icterus, cyanosis, clubbing, pedal edema or lymph node enlargement were noticed. Multiple cutaneous nodules of varying size were seen distributed on the entire body. Multiple *café au lait* pigmentation was noticed on the axillary region and on the arms. A large pigmented macule was present on the right side chest [Figure 2].

**Figure 6:**
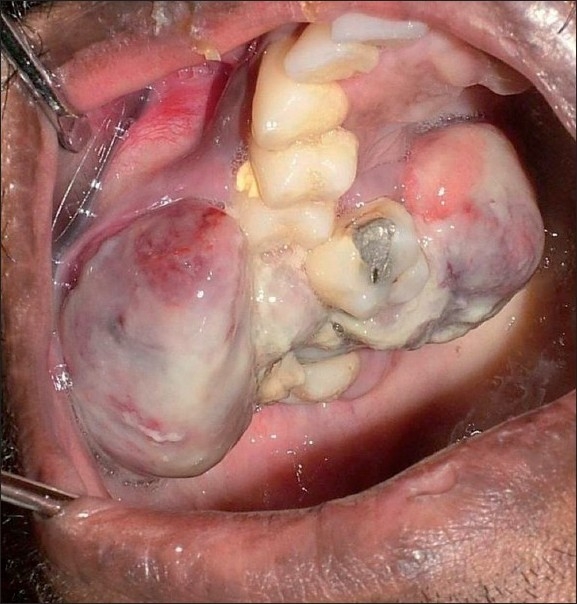
Lobulated, dumbbell shaped mass extending into the buccal vestibule and the palate, causing displacement of 16

On intraoral examination, an exophytic soft tissue mass measuring around 3 cm × 4 cm × 5 cm was present on the right alveolus in relation to 16 and 17. The lesion presented as a lobulated dumbbell shaped mass extending buccally and palatally. The buccal mass was found to be extending into the buccal vestibule, and the palatal mass involved the entire half of the posterior palate. The swelling was sessile, irregular in shape and normal in color. The surface was smooth with superficial candidal infection in some areas [Figure 6]. On palpation, the swelling was nontender, firm in consistency and was found to be fixed to the underlying tissue. Slight bleeding was noticed. Grade II mobility was present in 16 and 17.

**Figure 7:**
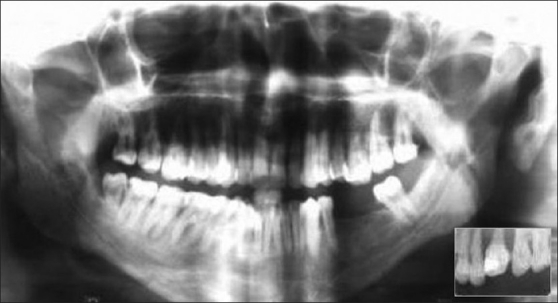
Orthopantamograph and IOPA radiograph showing bone loss in relation to 16 and 17

Intraoral periapical radiograph and orthopantamograph showed severe bone loss in relation to 16 and 17 with periapical radiolucency in relation to 16 [Figure 7].

Based on the history and clinical examination, a provisional diagnosis of intraoral neurofibroma was given. Other diagnosis considered included MPNST, other mesenchymal neoplasms and odontogenic neoplasms.

**Figure 8:**
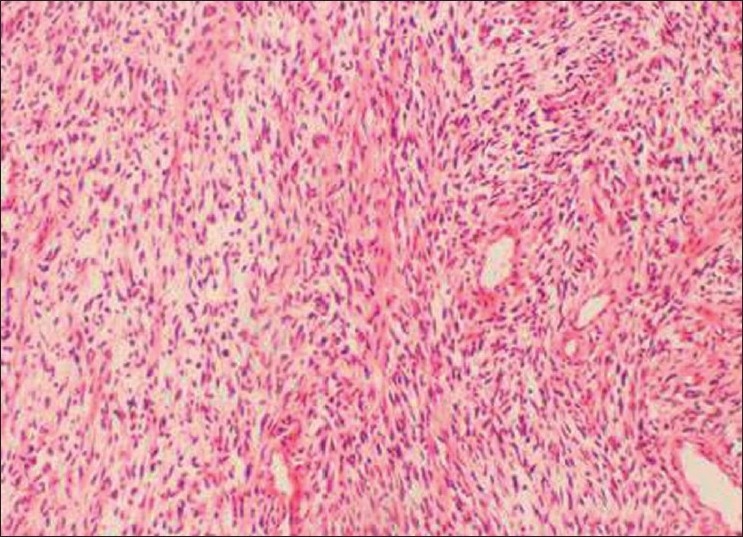
Alternating fascicles of hypercellular and hypocellular areas arranged in a streaming pattern (H and E, 20×)

**Figure 9:**
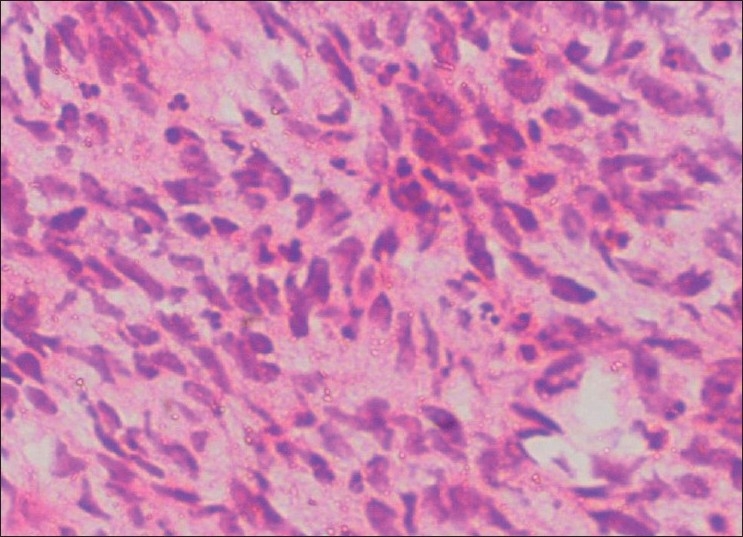
Atypical spindle cells showing pleomorphic, wavy nuclei and indistinct cytoplasm (H and E, 40×)

**Figure 10:**
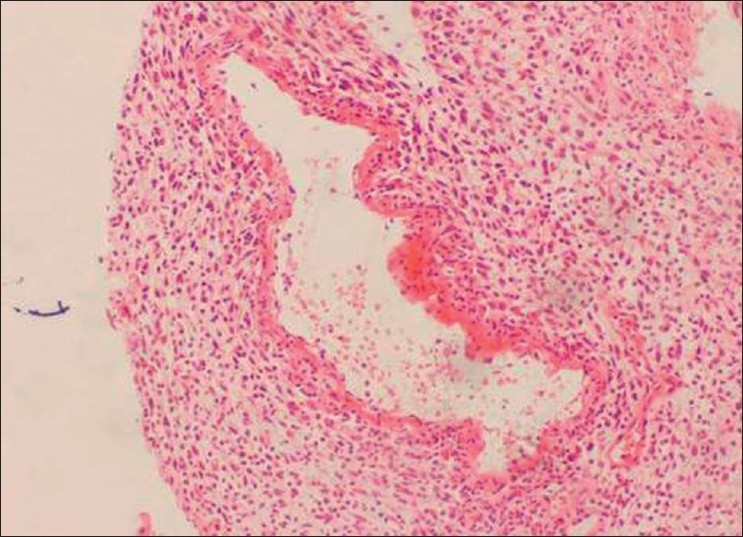
Vascular changes in MPNST. Sub-endothelial proliferation of tumor cells and herniation of tumor cells into the vessels (H and E, 40×)

**Figure 11:**
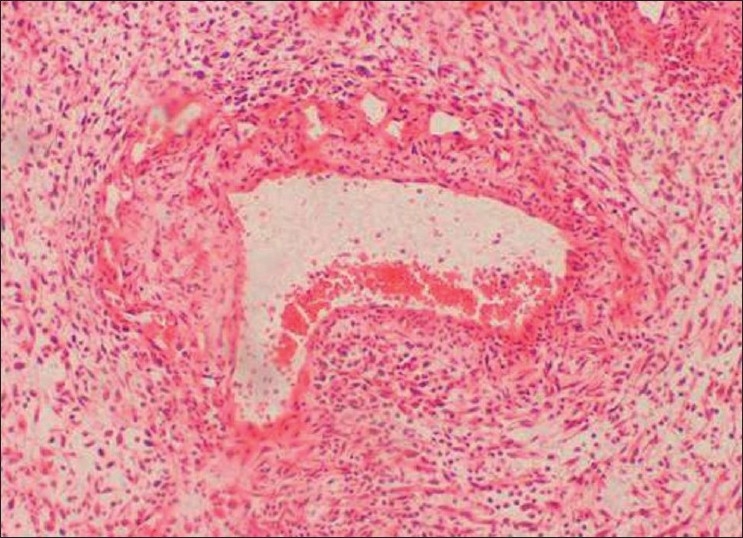
Vascular changes in MPNST. Proliferation of small vessels on the large vessel walls can be seen (H and E, 40×)

**Figure 12:**
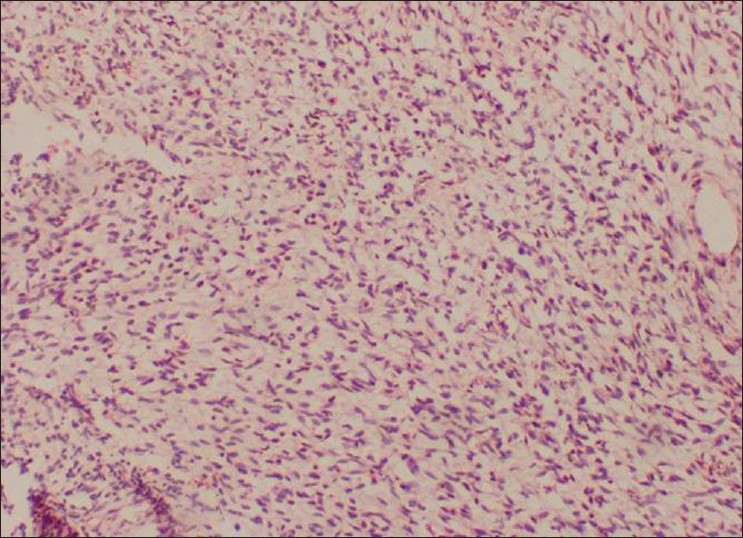
Areas resembling neurofibroma (H and E, 20×)

**Figure 13:**
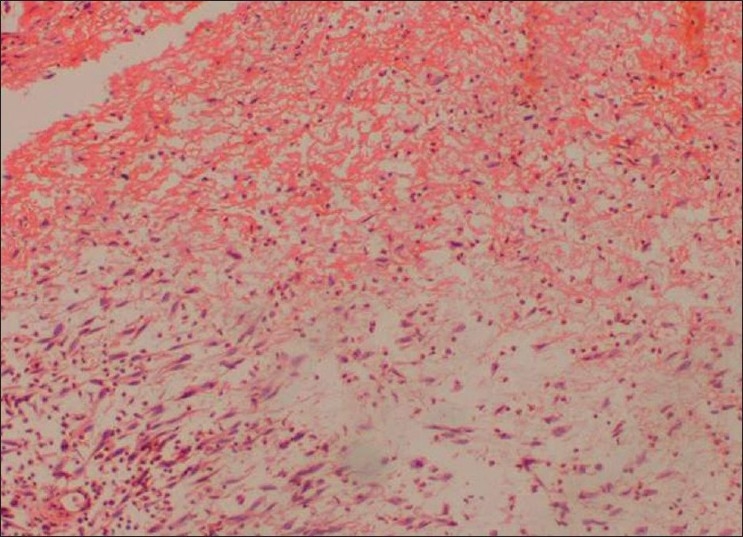
Areas of necrosis (H and E, 20×)

Incisional biopsy was done from the palatal aspect of the tumor. Microscopically, the lesion showed alternating fascicles of hypercellular and hypocellular areas arranged in a streaming pattern [Figure 8]. The cellular component comprised predominantly atypical spindle cells with hyperchromatic, wavy nuclei, which were pleomorphic, and indistinct cytoplasm [Figure 9]. Short fusiform cells with large hyperchromatic nuclei and a thin rim of cytoplasm were also seen. Increased mitosis (four to six per high power field) was noticed. The vascular changes like sub-endothelial proliferation of tumor cells and herniation of tumor cells into the vessels were appreciable in the sections [Figure 10]. The walls of some of the large vessels showed small vascular proliferations [Figure 11]. Neurofibromatous areas and areas of necrosis were also present [Figures 12 and 13]. Immunohistochemical staining by the tumor marker S-100 was found to be negative. Based on the histopathologic appearance and its clinical association with NF-1, the lesion was diagnosed as MPNST.

The patient was given radiotherapy for the oral lesion and was treated with 3000 cGy in 10 fractions. One month later, the patient developed similar lesions on the scalp and the right side of the upper back region and finally succumbed to death after 2 months.

## DISCUSSION

Neurofibromatosis, also known as VonRecklinghausen’s disease, named so after the person who described the disease in 1882, is an autosomal dominant disease of varied clinical manifestation. Two clinically and genetically distinct subtypes were identified and have been designated as NF-1 and NF-2.[3]

NF-1, otherwise known as the central form, is the most common single gene defect. It is clinically characterized by multiple neurofibromas along the peripheral nerves, optic gliomas, sphenoid wing dysplasias, pigmented iris nodules and hyperpigmented macular skin lesions known as *café au lait* spots.[4] All these manifestations may not be present always and the diagnostic criteria are met if the patient has two or more of the above-mentioned features.[5] The neurofibroma associated with NF-1 usually runs an indolent course but sometimes can undergo malignant transformation and in such cases can be fatal.

The peripheral nerve sheath tumors are the tumors arising from the nervous tissue outside the brain and the spinal cord. The term “malignant peripheral nerve sheath tumor” refers to all the spindle cell sarcomas arising from the peripheral nerve or neurofibroma or showing nerve sheath differentiation. Previously, various terminologies like neurogenic sarcoma, neurilemmosarcoma, malignant fibrosarcoma and malignant neurilemmoma had been used, but World Health Organization (WHO) has recently adopted the term “malignant peripheral nerve sheath tumor” to denote such tumors.[3] MPNSTs are highly aggressive tumors which make up around 10% of all soft tissue sarcomas. They are commonly seen in the extremities and trunk, in the deep soft tissue close to the distribution of sciatic nerve, branchial plexus and sacral plexus. Their occurrence in the head and neck region is extremely rare. Intraorally, MPNST may arise as a sporadic lesion or from a preexisting neurofibroma and very rarely may represent a metasatatic lesion. Clinically, they present as an enlarging mass, often associated with pain and nerve deficit. The tumors may occur anywhere in the oral cavity, but the most common sites are the mandible, lips and buccal mucosa.

Association of MPNST with NF-1 is well established. Around 40% of the MPNST cases were found to be associated with neurofibromatosis,[6] though among the patients with NF-1, the risk of developing MPNST is only 2–4%.[7] NF-1 is associated with deletion, insertion, or mutation in the *NF-1* gene, a tumor suppressor gene located on chromosome 17q11.2. This gene encodes a protein known as neurofibromin, which is believed to be important in the control of cell growth through their downregulation of *ras* gene product. Despite the fact that the precise chromosomal location of *NF-1* gene of chromosome 17 is known, neither the primary defect in *NF-1* nor the mechanism leading to malignant transformation is understood at the present time. Menon *et al*., based on their study, had proposed that an MPNST may result from several genetic hits. The first hit, a mutation in one or both copies of *NF-1* gene, may lead to the formation of a benign neurofibroma. Additional genetic hits leading to the loss of one or both copies of tumor suppressor gene (*p53* gene) on chromosome 17p may be required for the subsequent malignant transformation of a benign tumor.[8]

The diagnosis of MPNST is considered to be the most difficult and elusive in the soft tissue disease due to lack of standardized criteria.[3] The clinical findings need to be supplemented by the gross finding of a fusiform tumor in relation to a nerve, histopathology and immunohistochemistry for a conclusive diagnosis.[9] But it is not always possible to demonstrate the origin from a nerve, especially when it arises from a small peripheral branch.[10] The reliability of S-100 protein as the universal diagnostic marker for MPNST is also questionable as 30% of the cases may not show the focal positive reaction which is thought to be diagnostic for MPNST[11] as was observed in our case.

Diagnosis of MPNST by histopathologic analysis is often complicated by its resemblance to other spindle cell sarcomas like fibrosarcoma, malignant fibrous histiocytoma, leiomyosarcoma and monophasic synovial sarcoma. However, certain histological features which are characteristic of MPNST (summarized in Table 1)[3,12] may help us in differentiating MPNST from other spindle cell sarcomas, especially when it occurs as an isolated lesion. Though in our case the diagnosis was made easier by the histopathologic presentation of spindle cell sarcoma in the clinical setting of NF-1, the salient histopathologic criteria mentioned in the table were also met with.

**Table 1:** Histopathologic diagnostic criteria for MPNST

Pattern
Sweeping fascicles of spindle cells with densely cellular fascicles alternating with hypocellular myxoid zones Nodular, curlicue or whorled arrangement of spindle cells Heterogeneous differentiation

Cellular features

Atypical spindle cells with irregular contour Wavy, buckled or comma-shaped nuclei Cytoplasm usually indistinct and lightly stained

Subtle features characteristic of MPNST

Neoplastic cells appear to herniate into the lumen due to the eculiar proliferation of tumor cells in the sub-endothelial zone of vessels Proliferation of small vessels in the walls of large vessels Presence of neurofibromatous component (more evident in patients with NF-1) Geographic necrosis with pseudopalisading (this characteristic pattern of necrosis results from the persistence of viable perivascular cuffs of tumor cells)

MPNSTs are locally invasive lesions, frequently leading to multiple recurrences and eventual metastasic spread.[13] This tumor can spread through direct extension, hematogenous extension and by perineural spread. Lymph node metastasis is rare.[7] Most common metastatic sites are lungs, followed in decreasing order of frequency by soft tissue and bone. Very rarely, multifocal MPNSTs may occur in patients with NF-1. Our patient had multiple soft tissue lesions involving the mediastinum, oral mucosa, scalp and upper back region. The occurrence of multiple lesions can be attributed to either multifocality of the lesion or metastasis to distant site, the exact nature of which could not be established. But the absence of secondaries as seen in CT scan and MRI scan, and the presence of neurofibromatous components in the tissue sections are more in favor of a multifocal origin wherein some of the benign tumors might have undergone malignant transformation.

Prognosis of MPNST is poor and survival is found to be influenced by tumor location, size and association with NF-1. The presence of neurofibromatosis negatively affects many of the clinicopathological features of MPNST, and survival has been reported to be worse for patients with neurofibromatosis.[14] In our case, the malignancy developed after a period of 27 years, and following the initial diagnosis of MPNST, the patient survived hardly for another 6 months.

## CONCLUSION

NF-I may be considered as a familial cancer predisposition syndrome and the patients with NF-I need to be assessed periodically to rule out any malignant change. Histopathologic appearance of the lesional tissue still remains the mainstay of diagnosing MPNST, and careful and extensive microscopic study of the lesion may often give a conclusive diagnosis.
